# Supplementary material for: Age and Chronodisruption in Mouse Heart: Effect of the NLRP3 Inflammasome and Melatonin Therapy
Source: Int J Mol Sci. 2022 Jun 20;23(12):6846. doi: 10.3390/ijms23126846 (PMC9224376; doi:10.3390/ijms23126846)
Supplement: Supplementary file 1 [file ijms-23-06846-s001.zip › Supplementary Table S1.pdf]

Supplementary Table S1. Aging, genotype, melatonin effect on clock gene expression in WT and NLRP3$^{-/-}$ mice. Significant differences determined by *Tukey multiple comparison of mean* post-hoc test after Multifactorial-ANOVA.

Table S1.1. TRANSCRIPT: *Clock*. Post-hoc test: Tukey multiple comparison of mean

Aging effect on WT			***Genotype effect***			***Melatonin effect on WT***			***Melatonin effect on NLRP3$^{-/-}$***		
Hour	Comparison	P value	Hour	Comparison	P value	Hour	Comparison	P value	Hour	Comparison	P value
0	Y vs OA	<0.05	0	NLRP3$^{-/-}$ Y vs NLRP3$^{-/-}$ EA	<0.05	0	Y vs OA + aMT	<0.05	0	NLRP3$^{-/-}$Y vs NLRP3$^{-/-}$OA + aMT	<0.05
	EA vs OA	<0.05		NLRP3$^{-/-}$ Y vs NLRP3$^{-/-}$ OA	<0.05	6	Y vs OA + aMT	<0.05		NLRP3$^{-/-}$EA vs NLRP3$^{-/-}$EA + aMT	<0.05
6	Y vs EA	<0.05		NLRP3$^{-/-}$ EA vs NLRP3$^{-/-}$ OA	<0.05		EA vs EA + aMT	<0.05		NLRP3$^{-/-}$OA vs NLRP3$^{-/-}$OA + aMT	<0.05
	Y vs OA	<0.05		WT EA vs NLRP3$^{-/-}$EA	<0.05	12	Y vs OA + aMT	<0.05		WT EA + aMT vs NLRP3$^{-/-}$ EA + aMT	<0.05
	EA vs OA	<0.05		WT OA vs NLRP3$^{-/-}$ OA	<0.05		OA vs OA + aMT	<0.05	6	WT OA + aMT vs NLRP3$^{-/-}$ OA + aMT	<0.05
12	EA vs OA	<0.05	6	NLRP3$^{-/-}$ Y vs NLRP3$^{-/-}$ OA	<0.05	18	Y vs EA + aMT	<0.05	18	NLRP3$^{-/-}$ Y vs NLRP3$^{-/-}$ EA + aMT	<0.05
18	Y vs OA	<0.05		NLRP3$^{-/-}$ EA vs NLRP3$^{-/-}$ OA	<0.05		Y vs OA + aMT	<0.05		NLRP3$^{-/-}$ Y vs NLRP3$^{-/-}$ OA + aMT	<0.05
	EA vs OA	<0.05		WT EA vs NLRP3$^{-/-}$EA	<0.05		EA vs EA + aMT	<0.05		WT EA + aMT vs NLRP3$^{-/-}$ EA + aMT	<0.05
			12	NLRP3$^{-/-}$ Y vs NLRP3$^{-/-}$ EA	<0.05		OA vs OA + aMT	<0.05			
				NLRP3$^{-/-}$ EA vs NLRP3$^{-/-}$ OA	<0.05						
			18	NLRP3$^{-/-}$ Y vs NLRP3$^{-/-}$ EA	<0.05						
				NLRP3$^{-/-}$ Y vs NLRP3$^{-/-}$ OA	<0.05						
				NLRP3$^{-/-}$ EA vs NLRP3$^{-/-}$ OA	<0.05						

Table S1.2. TRANSCRIPT: *Bmal1*. Post-hoc test: Tukey multiple comparison of mean

Aging effect on WT			***Genotype effect***			***Melatonin effect on WT***			***Melatonin effect on NLRP3$^{-/-}$***		
Hour	Comparison	P value	Hour	Comparison	P value	Hour	Comparison	P value	Hour	Comparison	P value
0	Y vs EA	<0.001	0	NLRP3$^{-/-}$ Y vs NLRP3$^{-/-}$ EA	<0.001	0	Y vs EA + aMT	<0.001	0	NLRP3$^{-/-}$ Y vs NLRP3$^{-/-}$OA + aMT	<0.001
	Y vs OA	<0.001		NLRP3$^{-/-}$ Y vs NLRP3$^{-/-}$ OA	<0.001		Y vs OA + aMT	<0.001		WT EA + aMT vs NLRP3$^{-/-}$ EA + aMT	<0.001
6	Y vs EA	<0.001	6	WT Y vs NLRP3$^{-/-}$ Y	<0.01	6	Y vs EA + aMT	<0.05	12	NLRP3$^{-/-}$ Y vs NLRP3$^{-/-}$OA + aMT	<0.001
	EA vs OA	<0.05	12	NLRP3$^{-/-}$ EA vs NLRP3$^{-/-}$ OA	<0.05		Y vs OA + aMT	<0.05		NLRP3$^{-/-}$ OA vs NLRP3$^{-/-}$OA + aMT	<0.001
18	Y vs EA	<0.001	18	NLRP3$^{-/-}$ Y vs NLRP3$^{-/-}$ EA	<0.001	18	Y vs EA + aMT	<0.001	18	NLRP3$^{-/-}$ Y vs NLRP3$^{-/-}$EA + aMT	<0.05
	Y vs OA	<0.001		NLRP3$^{-/-}$Y vs NLRP3$^{-/-}$OA	<0.001		Y vs OA + aMT	<0.001		NLRP3$^{-/-}$ Y vs NLRP3$^{-/-}$OA + aMT	<0.001
				WT Y vs NLRP3$^{-/-}$ Y	<0.001		EA vs EA+aMT	<0.001		WT EA + aMT vs NLRP3$^{-/-}$ EA + aMT	<0.001
							EA + aMT vs OA	<0.001			
							EA + aMT vs OA + aMT	<0.001			

Table S1.3. TRANSCRIPT: *Per2*. Post-hoc test: Tukey multiple comparison of mean

Aging effect on WT

Hour	Comparison	P value
0	Y vs EA	<0.05
	EA vs OA	<0.05
6	Y vs OA	<0.05
	EA vs OA	<0.05
18	Y vs EA	<0.05
	Y vs OA	<0.05
	EA vs OA	<0.05

Genotype effect

Hour	Comparison	P value
0	NLRP3$^{-/-}$ Y vs NLRP3$^{-/-}$ OA	<0.05
	NLRP3$^{-/-}$ EA vs NLRP3$^{-/-}$ OA	<0.05
	WT EA vs NLRP3$^{-/-}$EA	<0.05
	WT OA vs NLRP3$^{-/-}$ OA	<0.05
6	NLRP3$^{-/-}$ Y vs NLRP3$^{-/-}$ EA	<0.05
	NLRP3$^{-/-}$ EA vs NLRP3$^{-/-}$ OA	<0.05
	WT EA vs NLRP3$^{-/-}$EA	<0.05
12	NLRP3$^{-/-}$ Y vs NLRP3$^{-/-}$ EA	<0.05
	NLRP3$^{-/-}$ Y vs NLRP3$^{-/-}$ OA	<0.05
18	NLRP3$^{-/-}$ Y vs NLRP3$^{-/-}$ OA	<0.05
	NLRP3$^{-/-}$ EA vs NLRP3$^{-/-}$ OA	<0.05
	WT Y vs NLRP3$^{-/-}$ Y	<0.05
	WT EA vs NLRP3$^{-/-}$EA	<0.05
	WT OA vs NLRP3$^{-/-}$ OA	<0.05

Melatonin effect on WT

Hour	Comparison	P value
6	Y vs EA + aMT	<0.05
	OA vs OA + aMT	<0.05
12	Y vs EA + aMT	<0.05
	EA vs EA + aMT	<0.05
18	Y vs EA + aMT	<0.05
	Y vs OA + aMT	<0.05
	EA vs EA + aMT	<0.05
	OA vs OA + aMT	<0.05

Melatonin effect on NLRP3$^{-/-}$

Hour	Comparison	P value
0	NLRP3$^{-/-}$ Y vs NLRP3$^{-/-}$ EA + aMT	<0.05
	NLRP3$^{-/-}$EA vs NLRP3$^{-/-}$EA + aMT	<0.05
	NLRP3$^{-/-}$OA vs NLRP3$^{-/-}$OA + aMT	<0.05
6	NLRP3$^{-/-}$ Y vs NLRP3$^{-/-}$EA + aMT	<0.05
	WT OA + aMT vs NLRP3$^{-/-}$ OA + aMT	<0.05
12	NLRP3$^{-/-}$EA vs NLRP3$^{-/-}$EA + aMT	<0.05
	NLRP3$^{-/-}$OA vs NLRP3$^{-/-}$OA + aMT	<0.05
	WT Y vs NLRP3$^{-/-}$Y	<0.05
	WT EA vs NLRP3$^{-/-}$EA	<0.05
	WT OA vs NLRP3$^{-/-}$ OA	<0.05
	WT EA + aMT vs NLRP3$^{-/-}$ EA + aMT	<0.05
	WT OA + aMT vs NLRP3$^{-/-}$ OA + aMT	<0.05
18	NLRP3$^{-/-}$ Y vs NLRP3$^{-/-}$EA + aMT	<0.05
	NLRP3$^{-/-}$OA vs NLRP3$^{-/-}$OA + aMT	<0.05
	WT EA + aMT vs NLRP3$^{-/-}$ EA + aMT	<0.05
	WT OA + aMT vs NLRP3$^{-/-}$ OA + aMT	<0.05

Table S1.4. TRANSCRIPT: *Chrono*. Post-hoc test: Tukey multiple comparison of mean

Aging effect on WT			***Genotype effect***			***Melatonin effect on WT***			***Melatonin effect on NLRP3$^{-/-}$***		
Hour	Comparison	P value	Hour	Comparison	P value	Hour	Comparison	P value	Hour	Comparison	P value
6	Y vs EA	<0.05	0	NLRP3$^{-/-}$ Y vs NLRP3$^{-/-}$ EA	<0.05	0	Y vs EA + aMT	<0.05	0	NLRP3$^{-/-}$ Y vs NLRP3$^{-/-}$ EA + aMT	<0.05
	Y vs OA	<0.05		NLRP3$^{-/-}$ Y vs NLRP3$^{-/-}$ OA	<0.05		Y vs OA + aMT	<0.05		NLRP3$^{-/-}$ Y vs NLRP3$^{-/-}$ OA + aMT	<0.05
	EA vs OA	<0.05		WT Y vs NLRP3$^{-/-}$ Y	<0.05		EA vs EA + aMT	<0.05		NLRP3$^{-/-}$ EA vs NLRP3$^{-/-}$ EA + aMT	<0.05
12	Y vs EA	<0.05		WT EA vs NLRP3$^{-/-}$ EA	<0.05		OA vs OA + aMT	<0.05		NLRP3$^{-/-}$ OA vs NLRP3$^{-/-}$ OA + aMT	<0.05
	Y vs OA	<0.05		WT OA vs NLRP3$^{-/-}$ OA	<0.05	6	Y vs EA + aMT	<0.05		WT EA + aMT vs NLRP3$^{-/-}$ EA + aMT	<0.05
	EA vs OA	<0.05	6	NLRP3$^{-/-}$ Y vs NLRP3$^{-/-}$ EA	<0.05		Y vs OA + aMT	<0.05		WT OA + aMT vs NLRP3$^{-/-}$ OA + aMT	<0.05
18	Y vs OA	<0.05		NLRP3$^{-/-}$ Y vs NLRP3$^{-/-}$ OA	<0.05		EA vs EA + aMT	<0.05	6	NLRP3$^{-/-}$ Y vs NLRP3$^{-/-}$EA + aMT	<0.05
	EA vs OA	<0.05		WT EA vs NLRP3$^{-/-}$ EA	<0.05	12	Y vs EA + aMT	<0.05		NLRP3$^{-/-}$ Y vs NLRP3$^{-/-}$OA + aMT	<0.05
				WT OA vs NLRP3$^{-/-}$ OA	<0.05		Y vs OA + aMT	<0.05		NLRP3$^{-/-}$ EA vs NLRP3$^{-/-}$ EA + aMT	<0.05
			12	NLRP3$^{-/-}$ Y vs NLRP3$^{-/-}$ EA	<0.05		EA vs EA + aMT	<0.05	12	NLRP3$^{-/-}$ Y vs NLRP3$^{-/-}$ EA + aMT	<0.05
				NLRP3$^{-/-}$ Y vs NLRP3$^{-/-}$ OA	<0.05		OA vs OA + aMT	<0.05		NLRP3$^{-/-}$ EA vs NLRP3$^{-/-}$ EA + aMT	<0.05
				WT Y vs NLRP3$^{-/-}$ Y	<0.05	18	Y vs EA + aMT	<0.05		NLRP3$^{-/-}$ OA vs NLRP3$^{-/-}$ OA + aMT	<0.05
				WT EA vs NLRP3$^{-/-}$ EA	<0.05		Y vs OA + aMT	<0.05		WT EA + aMT vs NLRP3$^{-/-}$ EA + aMT	<0.05
				WT OA vs NLRP3$^{-/-}$ OA	<0.05		EA vs EA + aMT	<0.05		WT OA +aMT vs NLRP3$^{-/-}$ OA +aMT	<0.05

Table S1.4. TRANSCRIPT: *Chrono*. Post-hoc test: Tukey multiple comparison of mean (continued)											
Aging effect on WT			***Genotype effect***			***Melatonin effect on WT***			***Melatonin effect on NLRP3$^{-/-}$***		
Hour	Comparison	P value	Hour	Comparison	P value	Hour	Comparison	P value	Hour	Comparison	P value
			18	NLRP3$^{-/-}$ Y vs NLRP3$^{-/-}$ OA	<0.05	18	OA vs OA + aMT	<0.05	18	NLRP3$^{-/-}$ Y vs NLRP3$^{-/-}$ OA + aMT	<0.05
				NLRP3$^{-/-}$ EA vs NLRP3$^{-/-}$ OA	<0.05					NLRP3$^{-/-}$ OA vs NLRP3$^{-/-}$ OA + aMT	<0.05
				WT Y vs NLRP3$^{-/-}$ Y	<0.05					WT EA + aMT vs NLRP3$^{-/-}$ EA + aMT	<0.05
				WT EA vs NLRP3$^{-/-}$ EA	<0.05					WT OA + aMT vs NLRP3$^{-/-}$ OA + aMT	<0.05
				WT OA vs NLRP3$^{-/-}$ OA	<0.05						

Table S1.5. TRANSCRIPT: *Rev-erba*. Post-hoc test: Tukey multiple comparison of mean											
Aging effect on WT			***Genotype effect***			***Melatonin effect on WT***			***Melatonin effect on NLRP3$^{-/-}$***		
Hour	Comparison	P value	Hour	Comparison	P value	Hour	Comparison	P value	Hour	Comparison	P value
0	Y vs EA	<0.05	0	NLRP3$^{-/-}$ Y vs NLRP3$^{-/-}$ EA	<0.05	6	Y vs EA + aMT	<0.05	6	NLRP3$^{-/-}$ Y vs NLRP3$^{-/-}$EA + aMT	<0.05
	EA vs OA	<0.05		NLRP3$^{-/-}$ EA vs NLRP3$^{-/-}$ OA	<0.05		Y vs OA + aMT	<0.05		NLRP3$^{-/-}$ Y vs NLRP3$^{-/-}$OA + aMT	<0.05
6	Y vs EA	<0.05	6	NLRP3$^{-/-}$ Y vs NLRP3$^{-/-}$ EA	<0.05	12	Y vs EA + aMT	<0.05	12	NLRP3$^{-/-}$ Y vs NLRP3$^{-/-}$EA + aMT	<0.05
	Y vs OA	<0.05		NLRP3$^{-/-}$ Y vs NLRP3$^{-/-}$ OA	<0.05		Y vs OA + aMT	<0.05		NLRP3$^{-/-}$ Y vs NLRP3$^{-/-}$OA + aMT	<0.05
12	Y vs EA	<0.05	12	NLRP3$^{-/-}$ Y vs NLRP3$^{-/-}$ EA	<0.05		EA vs EA + aMT	<0.05		NLRP3$^{-/-}$ EA vs NLRP3$^{-/-}$EA + aMT	<0.05
	Y vs OA	<0.05		NLRP3$^{-/-}$ Y vs NLRP3$^{-/-}$ OA	<0.05		OA vs OA + aMT	<0.05		NLRP3$^{-/-}$ OA vs NLRP3$^{-/-}$OA + aMT	<0.05
	EA vs OA	<0.05		NLRP3$^{-/-}$ EA vs NLRP3$^{-/-}$ OA	<0.05	18	Y vs EA + aMT	<0.05	18	NLRP3$^{-/-}$ Y vs NLRP3$^{-/-}$EA + aMT	<0.05
							Y vs OA + aMT	<0.05		NLRP3$^{-/-}$ Y vs NLRP3$^{-/-}$OA + aMT	<0.05
							EA vs EA + aMT	<0.05		NLRP3$^{-/-}$ EA vs NLRP3$^{-/-}$EA + aMT	<0.05

Table S1.6. TRANSCRIPT: *Rora*. Post-hoc test: Tukey multiple comparison of mean

Aging effect on WT			***Genotype effect***			***Melatonin effect on WT***			***Melatonin effect on NLRP3$^{-/-}$***		
Hour	Comparison	P value	Hour	Comparison	P value	Hour	Comparison	P value	Hour	Comparison	P value
0	Y vs OA	<0.05	0	NLRP3$^{-/-}$ Y vs NLRP3$^{-/-}$ EA	<0.05	0	Y vs EA + aMT	<0.05	0	NLRP3$^{-/-}$ Y vs NLRP3$^{-/-}$EA + aMT	<0.05
6	Y vs OA	<0.05		NLRP3$^{-/-}$ Y vs NLRP3$^{-/-}$ OA	<0.05		OA vs OA + aMT	<0.05		NLRP3$^{-/-}$ Y vs NLRP3$^{-/-}$OA + aMT	<0.05
	EA vs OA	<0.05		WT Y vs NLRP3$^{-/-}$ Y	<0.05	6	Y vs OA + aMT	<0.05	6	NLRP3$^{-/-}$ Y vs NLRP3$^{-/-}$EA + aMT	<0.05
18	EA vs OA	<0.05	6	NLRP3$^{-/-}$ Y vs NLRP3$^{-/-}$ EA	<0.05	12	Y vs OA + aMT	<0.05		NLRP3$^{-/-}$ Y vs NLRP3$^{-/-}$OA + aMT	<0.05
				NLRP3$^{-/-}$ Y vs NLRP3$^{-/-}$ OA	<0.05		OA vs OA + aMT	<0.05		NLRP3$^{-/-}$ EA vs NLRP3$^{-/-}$ EA + aMT	<0.05
				NLRP3$^{-/-}$ EA vs NLRP3$^{-/-}$ OA	<0.05	18	Y vs EA + aMT	<0.05	12	NLRP3$^{-/-}$ EA vs NLRP3$^{-/-}$ EA + aMT	<0.05
				WT Y vs NLRP3$^{-/-}$ Y	<0.05		Y vs OA + aMT	<0.05		NLRP3$^{-/-}$ OA vs NLRP3$^{-/-}$ OA + aMT	<0.05
				WT EA vs NLRP3$^{-/-}$ EA	<0.05		EA vs EA + aMT	<0.05		WT OA + aMT vs NLRP3$^{-/-}$ OA + aMT	<0.05
				WT OA vs NLRP3$^{-/-}$ OA	<0.05		OA vs OA + aMT	<0.05	18	NLRP3$^{-/-}$ Y vs NLRP3$^{-/-}$EA + aMT	<0.05
			12	NLRP3$^{-/-}$ Y vs NLRP3$^{-/-}$ EA	<0.05					NLRP3$^{-/-}$ Y vs NLRP3$^{-/-}$OA + aMT	<0.05
				NLRP3$^{-/-}$ Y vs NLRP3$^{-/-}$ OA	<0.05					NLRP3$^{-/-}$ EA vs NLRP3$^{-/-}$ EA + aMT	<0.05
				NLRP3$^{-/-}$ EA vs NLRP3$^{-/-}$ OA	<0.05					WT EA + aMT vs NLRP3$^{-/-}$ EA + aMT	<0.05
				WT EA vs NLRP3$^{-/-}$ EA	<0.05					WT OA + aMT vs NLRP3$^{-/-}$ OA + aMT	<0.05
			18	NLRP3$^{-/-}$Y vs NLRP3$^{-/-}$OA	<0.05						
				NLRP3$^{-/-}$EA vs NLRP3$^{-/-}$OA	<0.05						
				WT Y vs NLRP3$^{-/-}$ Y	<0.05						
				WT EA vs NLRP3$^{-/-}$ EA	<0.05						
