# Supplementary material for: Age and Chronodisruption in Mouse Heart: Effect of the NLRP3 Inflammasome and Melatonin Therapy
Source: Int J Mol Sci. 2022 Jun 20;23(12):6846. doi: 10.3390/ijms23126846 (PMC9224376; doi:10.3390/ijms23126846)
Supplement: Supplementary file 1 [file ijms-23-06846-s001.zip › Supplementary Table S2.pdf]

Supplementary Table S2. Cosinor analysis of clock gene expression in heart of WT and NLRP3$^{-/-}$ mice during aging and melatonin treatment.

Group	Gene	P-value	PR	Acrophase (95% CI)	Amplitude (95% CI)	Mesor (95% CI)
WT Y	*Clock*	< 0.001	83,31	7.53 (6.21 – 8.87)	20 (13.30 - 26.80)	69.2 (64.50 - 74)
	Bmal1	< 0.001	73,48	7.67 (6.62 – 8.87)	28.60 (20.80 – 36.40)	50.80 (45.30 – 56.30)
	Per2	< 0.01	35,83	19.87 (17.40 – 22.40)	10.40 (4.07 – 16.30)	45.60 (41.20 – 50.10)
	Chrono	< 0.05	63,17	19.67 (17.33 - 22)	8.96 (3.80 – 14.10)	22.10 (18.40 – 25.70)
	Rev-erbα	< 0.001	65,24	16.73 (15.47 - 18)	27.20 (18.20 – 36.20)	21.70 (15.30 – 28.10)
	Rorα	< 0.001	83,25	13.33 (12.47 -14.20)	15.50 (12 – 19.10)	53.20 (50.70 – 55.70)
NLRP3$^{-/-}$ Y	*Clock*	< 0.05	48,22	7.80 (5.26 – 10.40)	7.60 (2.80 – 12.30)	55.90 (52.50 – 59.20)
	Bmal1	< 0.01	43,63	7.93 (5.84 - 10)	17.30 (8.37 – 26.20)	30.50 (24.20 – 36.90)
	Per2	< 0.05	58,63	12.47 (9.87 – 15.13)	19.70 (7.20 – 32.10)	53.80 (45 – 62.70)
	Chrono	< 0.001	81,06	17.73 (16.87 – 18.53)	34.90 (27.20 – 42.50)	45.80 (40.40 – 51.30)
	Rev-erbα	< 0.001	72,8	17.40 (16.33 – 18.47)	35.70 (25.80 – 45.70)	28.40 (21.40 – 35.40)
	Rorα	< 0.05	58,83	18.01 (15.47 – 20.67)	26.50 (9.79 – 43.30)	74.30 (62.40 – 86.10)
WT EA	*Clock*	< 0.01	37,08	12.20 (9.80 – 14.60)	14.70 (6.03 – 23.50)	60.60 (54.50 – 66.80)
	Bmal1	< 0.001	72,58	10.87 (9.80 - 11.93)	34.90 (25.20 – 44.70)	26.90 (20 – 33.80)
	Per2	< 0.05	61,46	21.33 (18.87 – 23.73)	23.80 (9.58 - 38)	58.70 (48.60 – 68.70)
	Chrono	< 0.001	86,97	17.27	14.80	22.70

				(16.60 - 17.93)	(12.20 – 17.40)	(20.90 – 24.50)
	Rev-erbα	< 0.001	80,37	16.07<br>(15.20 - 16.93)	24.40<br>(18.90 – 29.80)	30.80<br>(26.90 – 34.60)
	Rorα	ns	23,86	13.80<br>(10.20 – 17.40)	13.20<br>(2.49 – 23.90)	54.50<br>(47 – 62.10)
**NLRP3$^{-/-}$ EA**	***Clock***	< 0.001	80,06	10.87<br>(10 - 11.73)	29.80<br>(23 – 36.50)	67.60<br>(62.90 – 72.40)
	Bmal1	< 0.001	74,63	8.80<br>(7.73 – 9.80)	26.50<br>(19.50 – 33.50)	24.70<br>(19.80 – 29.70)
	Per2	< 0.001	52,92	11.13<br>(9.47 – 12.80)	12.80<br>(7.32 – 18.30)	56.10<br>(52.20 – 59.90)
	Chrono	< 0.001	65,24	17.73<br>(16.47 – 19.07)	34.90<br>(23.30 – 46.50)	24.60<br>(16.50 – 32.80)
	Rev-erbα	< 0.001	95,62	15.07<br>(14.67 – 15.40)	43.60<br>(39.40 – 47.90)	36.90<br>(33.90 – 39.90)
	Rorα	ns	3,03	19.40<br>(16.27 – 22.53)	4.28<br>(-6.71 – 15.30)	56.80<br>(49 – 64.60)
**WT EA + aMT**	***Clock***	< 0.001	78,5	7.27<br>(6.34 – 8.20)	29.50<br>(22.50 – 36.50)	50.90<br>(45.90 – 55.90)
	Bmal1	< 0.001	96,16	9.33<br>(8.93 – 9.67)	28.90<br>(26.30 -31.60)	22.70<br>(20.80 – 24.50)
	Per2	< 0.01	43,64	6.65<br>(4.58 – 8.73)	15.50<br>(7.50 – 23.50)	47.60<br>(41.90 – 53.30)
	Chrono	< 0.001	77,88	20.60<br>(19.67 – 21.53)	4.73<br>(3.59 – 5.87)	9.55<br>(8.74 – 10.40)
	Rev-erbα	< 0.001	67,33	12.13<br>(10.93 – 13.40)	40.40<br>(27.60 – 53.10)	31.50<br>(22.50 – 40.60)
	Rorα	< 0.05	54,57	6.63<br>(3.73 – 9.53)	23<br>(7.17 – 38.80)	45<br>(33.80 – 56.20)
**NLRP3$^{-/-}$ EA + aMT**	***Clock***	< 0.01	46,18	6.48<br>(4.53 – 8.47)	13.90<br>(7.10 – 20.80)	68.80<br>(64 – 73.70)
	Bmal1	< 0.001	95,65	7.40<br>(7 - 7.73)	17.30<br>(15.60 - 19)	19.70<br>(18.50 – 20.90)
	Per2	< 0.05	41,03	10.80<br>(7.80 – 13.87)	12.30<br>(3.47 – 21.20)	60.90<br>(54.70 – 67.20)
	Chrono	< 0.001	66,5	18.27	32.10	22.80

				(17 – 19.53)	(21.80 – 42.50)	(15.50 – 30.10)
	Rev-erbα	< 0.001	90,93	15.60<br>(15 – 16.13)	40.20<br>(34.40 – 45.90)	36.60<br>(32.50 – 40.70)
	Rorα	< 0.05	40,9	13.13<br>(10.07 – 16.20)	9.46<br>(2.65 – 16.30)	56.60<br>(51.70 – 61.40)
**WT**<br>**OA**	***Clock***	< 0.01	46,41	11.40<br>(9.40 – 13.33)	13.10<br>(6.73 – 19.50)	41.90<br>(37.40 – 46.50)
	Bmal1	< 0.001	86,62	9.13<br>(8.40 - 9.80)	37<br>(30.40 – 43.60)	30.20<br>(25.50 – 34.90)
	Per2	ns	20,98	20.93<br>(16.80 – 25.07)	6.01<br>(0.72 – 11.30)	35<br>(31.20 – 38.70)
	Chrono	< 0.001	73,24	18.67<br>(17.60 – 19.73)	42.20<br>(30.70 – 53.80)	29.60<br>(21.40 – 37.80)
	Rev-erbα	< 0.001	77,94	15.73<br>(14.80 – 16.67)	21<br>(16 – 26.10)	24.50<br>(20.90 - 28)
	Rorα	ns	1,35	16<br>(12.93 – 19.07)	2.76<br>(-7.94 – 13.40)	65.60<br>(58 – 73.20)
**NLRP3$^{-/-}$**<br>**OA**	***Clock***	< 0.001	67,88	10.40<br>(9.20 – 11.60)	22.30<br>(15.40 – 29.30)	40.60<br>(35.60 – 45.50)
	Bmal1	< 0.001	83,88	9.80<br>(9 – 10.53)	41<br>(32.80 – 49.10)	32.70<br>(26.90 – 38.40)
	Per2	< 0.001	78,38	10.47<br>(9.53 – 11.33)	19.30<br>(14.70 – 23.90)	37.70<br>(34.50 - 41)
	Chrono	< 0.001	55,85	17.47<br>(15.87 – 19.07)	13<br>(7.74 – 18.20)	13.60<br>(9.86 – 17.30)
	Rev-erbα	< 0.001	70,96	16<br>(14.87 – 17.13)	25.40<br>(18 – 32.80)	22.40<br>(17.20 – 27.60)
	Rorα	ns	28,59	15.27<br>(11.20 – 19.40)	3.43<br>(-0.66 – 7.53)	51.70<br>(48.80 – 54.60)
**WT**<br>**OA + aMT**	***Clock***	< 0.01	38,43	7<br>(4.66 – 9.33)	9.11<br>(3.80 – 14.30)	36.20<br>(32.50 – 39.90)
	Bmal1	< 0.001	81,19	8.40<br>(7.53 – 9.27)	22.20<br>(17.40 – 27.10)	22<br>(18.60 – 25.40)
	Per2	< 0.001	50,67	4.63<br>(2.86 – 6.41)	13.50<br>(7.45 – 19.50)	36.50<br>(32.30 – 40.80)
	Chrono	< 0.001	66,99	19.80	7	10.70

				(18.40 – 21.20)	(4.48 – 9.51)	(8.92 – 12.50)
	Rev-erbα	< 0.001	93,08	13.93<br>(13.27 - 14.53)	16.40<br>(13.70 - 19)	20.60<br>(18.70 – 22.50)
	Rorα	< 0.001	79,2	6.01<br>(4.49 – 7.53)	22.80<br>(14 – 31.60)	36.30<br>(30.10 – 42.60)
**NLRP3$^{-/-}$ OA + aMT**	***Clock***	< 0.01	41,73	7.53<br>(5.37 – 9.67)	20.50<br>(9.25 – 31.50)	42.30<br>(34.50 – 50.10)
	Bmal1	< 0.001	86,25	7.40<br>(6.67 – 8.07)	16.10<br>(13.20 - 19)	15<br>(13 – 17.10)
	Per2	< 0.001	64,39	12.60<br>(11.33 – 13.93)	28.80<br>(19.10 – 38.50)	51.30<br>(44.40 – 58.20)
	Chrono	< 0.001	86,27	16.01<br>(15.13 - 17)	28.10<br>(21.40 – 34.80)	26<br>(21.30 – 30.80)
	Rev-erbα	< 0.001	84,81	14.33<br>(13.60 – 15.07)	41.90<br>(33.80 – 49.90)	35.60<br>(29.90 – 41.30)
	Rorα	< 0.001	82,95	11.20<br>(9.87 - 12.53)	31.10<br>(20.50 – 41.80)	60.80<br>(53.30 – 68.40)

[a]PR: percentage of rhythm; [b]CI: confidence interval
