# Supplementary material for: Age and Chronodisruption in Mouse Heart: Effect of the NLRP3 Inflammasome and Melatonin Therapy
Source: Int J Mol Sci. 2022 Jun 20;23(12):6846. doi: 10.3390/ijms23126846 (PMC9224376; doi:10.3390/ijms23126846)
Supplement: Supplementary file 1 [file ijms-23-06846-s001.zip › Supplementary Table S3.pdf]

Supplementary Table S3. List of primers used in RT-PCR assay.

Gene Symbol	Gene Description	Forward primer	Reverse primer
Clock	Circadian Locomotor Output Cycles Kaput	GGTGGTGACTGCCTATCCTAC	CTGCTGTTGTTGTTGCTGTTG
Bmal1	Brain and muscle ARNT (aryl hydrocarbon receptor nuclear translocator)-like 1	GAAGACAATGAGCCAGACAAC	CCATAGATTTCACCCGTATTTCC
Per2	Period circadian clock 2	ATCTATCTGTGCTGCTGGTC	ACTGGTGATGTCTCGTTCC
Chrono	Circadian associated repressor of transcription	GCATTGGTGTCATCCTTGTC	TTAGTCATCTCTCTGTCTGTGG
Rev-erbα	Reverse strand of protein ERB alpha	ACACACTCTCTGCTCTTC	GACCTTGACACAAACTGG
Rorα	Retinoic acid-related orphan receptor alpha	AGGTGGTGTTTATTAGGATGTG	TCTTCTCGGTGGTTCTTCT
β-actin	Beta-actin	GCTGTCCCTGTATGCCTCTG	CGCTCGTTGCCAATAGTGATG
